# Propionic acidemia as a framework for understanding the impact of disturbed propionyl-CoA on histone modifications

**DOI:** 10.1042/BST20260178

**Published:** 2026-08-27

**Authors:** Pawel Swietach, Eleanor K. Gill, Lorenz M.W. Holzner

**Affiliations:** 1 Department of Physiology, Anatomy & Genetics, University of Oxford, Oxford OX1 3PT, U.K.; 2 Epigenetics & Signalling Programmes, Babraham Institute, Cambridge CB22 3AT, U.K.

**Keywords:** Heart, Liver, Propionate, Propionic acidemia

## Abstract

Histone propionylation—the transfer of a three-carbon propionyl group from propionyl-CoA to lysine residues—is an emerging post-translational modification (PTM) with potential effects on gene expression. Interest in this PTM has grown because propionyl-CoA is at the interface between valine, isoleucine, methionine, and threonine catabolism, odd-chain fatty acid oxidation, gut-derived propionate assimilation, and anaplerotic entry into the tricarboxylic acid cycle. An informative context for studying the influence of disturbed propionyl-CoA metabolism on chromatin state and phenotype is propionic acidemia (PA), where loss-of-function mutations in genes coding propionyl-CoA carboxylase (PCC) lead to the accumulation of propiogenic substrates and propionyl-CoA. At least some of the clinical manifestations of PA (cardiomyopathy, arrhythmia, and neurological injury) may relate to altered histone propionylation, and a challenge is to distinguish these responses from metabolic toxicity. To that end, mouse models of PA are promising tools for connecting molecular-level histone changes to systems-level phenotypes. In this review, we describe recent advances in propionyl-CoA biology in the context of histone modifications and gene regulation. We present milestone studies that identified histone PTMs and described their association with phenotype. We also highlight emerging questions related to propionyl-CoA handling, its compartmentalization, and tissue specificity of actions, and discuss the relative contribution of histone propionylation versus other forms of acylation and broader metabolic stress. On balance, the literature supports a model in which propionyl-CoA exerts regulatory actions on chromatin state, but how this might be targeted therapeutically in PA and the implications for other metabolic disturbances remain important areas for further investigation.

## Introduction

**Figure 1:**
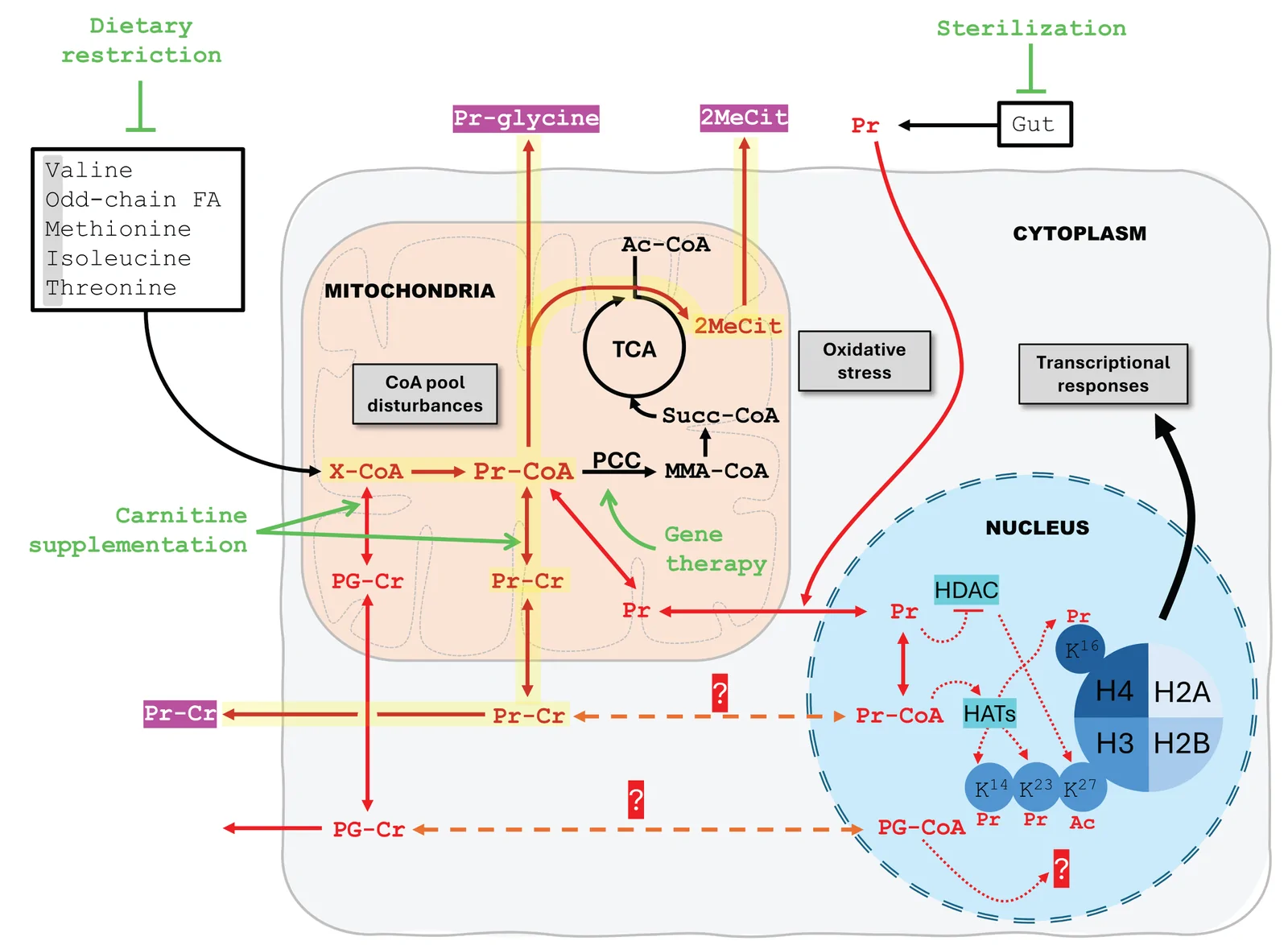
Schematic of selected metabolic pathways and signalling cascades related to propionate Arrows indicate pathways affected by the build-up of propionyl-CoA (Pr-CoA) in mitochondria (light red), cytoplasm (grey), and nucleus (light blue). Pr = propionate. Cr = carnitine. PG = propiogenic. 2MeCit = 2-methylcitrate. MMA = methylmalonyl. Succ = succinyl. Ac = Acetyl. PCC = propionyl-CoA carboxylase. HAT = histone acyltransferase. HDAC = histone deacetylase. Pathways highlighted in yellow indicate the canonical pathway disturbed in PA, including blood markers used diagnostically (2MeCit, Pr-Cr, and Pr-glycine in purple). Red lines indicate additional pathways relevant to histone modifications. Dashed orange lines indicate hypothetical routes for which evidence is indirect or lacking. Dotted red lines show processes that lead to histone modifications. Green arrows indicate therapeutic interventions.

Propionic acidemia (PA), although a rare genetic disorder, is among the most common organic acidemias [1] that has attracted growing research attention over the past decade, enabled by strong support from charities, including the Propionic Acidemia Foundation and the Organic Acidemia Association. The underlying biochemical deficiency is in propionyl-CoA carboxylation, a mitochondrial process that commits the 3-carbon propionyl moiety into the tricarboxylic acid (TCA) cycle as the 4-carbon moiety of succinyl-CoA via methylmalonyl-CoA (Figure 1; *pathway highlighted yellow*). Critically, this reaction is catalyzed solely by propionyl-CoA carboxylase (PCC), which explains why mutations in *PCCA* and *PCCB* genes are sufficient to produce the severe biochemical disturbances observed in PA. Primary consequences include the build-up of propionyl-CoA, propionylated derivatives (e.g. propionylcarnitine, N-propionylglycine), and condensation products of propionyl-CoA instead of acetyl-CoA (e.g. 2-methylcitrate) (Figure 1; *purple*). Secondary biochemical consequences include hyperammonemia when drainage of TCA intermediates reduces hepatic N-acetylglutamate synthesis necessary for the urea cycle and hyperglycinemia due to inhibition of glycine cleavage. However, the molecular mechanisms by which these disturbances cause clinical manifestations—such as cardiac complications, neurological problems, and developmental disability—remain incompletely understood. Consequently, current diagnostics for tracking disease progression and monitoring treatment responses prioritize measurements of biochemical readouts over readouts of processes more closely linked to clinical manifestations.

An important conceptual advance in linking disrupted propionyl-CoA with organ-level dysfunction was the recognition that propionyl-CoA can post-translationally modify proteins at lysine residues, including p53, p300, and CREB-binding protein [2], as well as histones [3]. Since histone PTMs can change chromatin structure and gene expression, significant efforts have been undertaken to link these to long-term PA pathophysiology [4–7]. This is an extension of a well-established principle that metabolism and epigenetics are coupled mechanistically, ostensibly to convey metabolic state into a transcriptional response [4,8]. The *in vivo* relevance of these histone PTMs was first demonstrated using a hypomorphic mouse model of PA developed by Michael Barry and colleagues [9]. This widely disseminated tool addressed the problem of severe lethality of *Pcca* knockout mice (death typically in first 36 h) by introducing a mutant human transgene (A138T) with 2% of wild-type PCC activity, sufficient to produce a PA-like metabolic disturbance whilst permitting survival to adulthood. A later-developed mouse model that circumvents neonatal lethality was the inducible *Pcca* knockout [7]. With widening access to PA mice, several questions have emerged: How is propionyl-CoA distributed in cells? How is histone propionylation regulated, and what is its relationship with other PTMs? How do these changes impact gene expression and phenotype? Can this knowledge be exploited therapeutically for PA, and what does it tell us about propionyl-CoA signaling more broadly?

This review highlights recent literature on histone propionylation and its role in PA, while recognizing that it is one of several mechanisms that may contribute to the disease phenotype. For a broader overview of the impact of metabolic toxicity, including oxidative stress, mitochondrial dysfunction, and altered CoA pools, we refer to recent review articles [10–14].

## Evidence for histone propionylation as a distinct post-translational modification

The first evidence that histone propionylation is biologically relevant came from studies outside PA-centered research. Chen et al. detected histone propionylation (and butyrylation) as novel PTMs, establishing short-chain acyl marks beyond acetylation [3]. Later, Liu et al. reported histone H3 lysine 23 (H3K23) propionylation in mammalian cells, demonstrating that this modification is not an artifact of *in vitro* chemical derivatization but a *bona fide* chromatin mark [15]. The bromodomain protein Brd4, canonically linked to reading acetylated lysine, was shown to recognize propionylated and butyrylated histone lysine marks, which indicated a mechanism by which these novel PTMs could influence transcription [16]. Collectively, these seminal studies progressed propionylation from a biochemical curiosity to a plausible epigenetic mechanism, but did not yet point to the metabolic source of the propionyl moiety. Insights into these supply lines were obtained from studying PA.

The discovery of histone H3 propionylation at lysine 14 (H3K14pr) in hypomorphic PA mouse liver was groundbreaking because it challenged the dogma that methylation and acetylation were the sole histone PTMs that link metabolism with epigenetics *in vivo* [4]. Since acetylation of this lysine residue had already been linked to transcriptional activation, alternative acyl modifications were proposed to arise from altered substrate availability for histone acyl-transferases (HATs). Indeed, the HATs GCN5 and PCAF were found to have propionyl-transferase activity *in vivo* [4]. The subsequent demonstration that lysine acetyltransferases 6A and 6B (KAT6A/B) can deposit propionylation at H3K23 [17] and that HAT1 contributes to H4K12 propionylation [18] argued that even canonical acetyl-transferases have a substrate spectrum beyond acetyl-CoA. This promiscuity in homologous substrate handling is consistent with the detection of methyl-substituted analogues of canonical metabolites, including 2-methylcitrate in place of citrate and 2-methylaconitate in place of aconitate in PA [19].

Studies reporting H3K14pr and H3K23pr found that these marks are enriched, genome-wide, at promoters of active genes, but whether chemically distinct acyl groups produce distinguishable transcriptional responses remained unclear. Evidence for modification-specific responses was obtained in a non-PA context for propionate and butyrate. In epithelial cells, genome-wide mapping of H3K18pr/H3K18bu and H4K12pr/H4K12bu showed that these short-chain acyl marks are associated with genomic regions distinct from their acetylated counterparts. Ingenious experiments using semi-synthetic nucleosomes provided a new level of precision in studying the actions of acyl-chain length on histone structure [5]. H4K16 modifications were studied on the basis that the tail of histone H4 makes direct contact with an acidic patch of neighboring nucleosomes, shaping chromatin compaction. Neutralization of the positive charge of H4K16 by acetylation was found to open chromatin and activate transcription, but the effect was distinguishable from that of propionylation and butyrylation, ostensibly due to differences in hydrophobicity. This observation demonstrated the potential for converting distinct metabolic signals into unique transcriptional responses, paving the way for further studies that unravel the depth of information carried by such modifications.

## Propionylation substrate access to nuclear histones: transport and compartmentalization

HATs require acyl-CoA esters to transfer the acyl moiety onto histone lysine residues, yet propionyl-CoA generated in the mitochondrial matrix cannot cross membranes due to its large size. The pathway that transfers acyl-CoAs between mitochondria and the nucleus is not fully mapped and may be tissue-specific, but its operation could introduce an additional layer of regulation resulting in subcellular compartmentalization (Figure 1). To access extra-mitochondrial cell compartments, propionyl-CoA can de-esterify to form free propionate, which crosses the lipid bilayer when protonated. Re-esterification by enzymes such as ACSS3 found in the nucleus can generate propionyl-CoA locally to modify histones [20]. Esterification could also be cytoplasmic, followed by diffusion across nuclear pores. A recent study showed that inactivating acyl-CoA synthase activity in the nucleus did not prevent histone PTMs [21], suggesting an alternative pathway linking mitochondrial and nuclear propionyl-CoA. Such a pathway may involve carnitine O-acetyltransferase, or similar enzyme, which converts propionyl-CoA to propionylcarnitine followed by translocation across the inner membrane by carnitine-acylcarnitine translocase. Once extra-mitochondrial, the carnitine derivative would require enzymatic activity to convert back to the acyl-CoA, but the source of this catalysis is currently unknown.

The indirect transfer of propionyl-CoA between mitochondria and the nucleus provides opportunities for subcellular compartmentalization, an outcome that would not be possible with highly permeable signals. Moreover, recent studies showed that propionyl-CoA can partition between cytoplasm and nucleoplasm [22], which is intriguing because these compartments are coupled through high-permeability nuclear pores. Standing acyl-CoA gradients are plausible when the dynamic equilibrium between esterification and hydrolysis involves spatially separated enzymes of distinct substrate specificity. For example, segregating propionyl-CoA-selective esterase activity in the cytoplasm and propionyl-CoA synthase activity in the nucleus is predicted to raise nuclear propionyl-CoA above cytoplasmic levels. Such shuttle systems could amplify (or attenuate) the delivery of propionyl-CoA to its nuclear site of action, but their physiological significance remains to be determined.

A noteworthy non-mitochondrial source of propionate is production by the gut microbiome, which can produce histone PTMs in intestinal epithelium [18] (Figure 1). Blood sampling at different points along the circulation of mice showed that germ-free conditions substantially reduced hepatic portal vein propionate [23]. In mice on normal chow but lacking sufficient PCC activity, the liver fails to clear hepatic portal venous blood from propionate, flooding the body with propionate and trapping propionyl-CoA in tissues characterized by a high CoA esterification rate and low anaplerosis to TCA, notably the heart [23].

## Scope for non-propionyl histone modifications under dysregulated propionate metabolism

When mitochondrial PCC activity is insufficient to commit propionyl-CoA to the TCA cycle, the build-up of matrix propionyl-CoA can disturb metabolism more broadly and may lead to additional PTMs. Free propionate released from propionyl-CoA may affect histones via inhibition of histone deacetylases (HDACs), as described originally for butyrate (Figure 1). This effect was inferred in PA hearts from the high degree of histone acetylation, notably as H3K27, that generally colocalized with propionylation marks at promoters of active genes [6]. Mitochondrial propionyl-CoA build-up can also disturb upstream acyl-CoAs that could follow a similar exit path to propionyl-CoA in reaching the nucleus (Figure 1). Indeed, a rise in propionyl-CoA is unlikely to be an isolated metabolic change because of the convergence of multiple pathways. For example, propionogenic substrates, summarized by the mnemonic VOMIT (valine, odd-chain fatty acids, methionine, isoleucine, and threonine), are catabolized via a number of CoA intermediates, which may accumulate in conditions such as PA. This is illustrated by the build-up of isobutyryl-CoA (downstream of valine), tiglyl-CoA, and 2-methylbutyryl-CoA (downstream of isoleucine) in the hearts of hypomorphic PA mice [6]. With evidence for histone crotonylation, succinylation, malonylation, and lactylation [3,7,8,24–27], there is scope for diversity in histone PTMs under disturbed propionate metabolism.

In summary, propionyl-CoA disturbances are unlikely to act through a single isolated histone mark; instead, epigenetic responses reflect the balance of various acylation events. The functional consequences of these marks are challenging to predict, and a first step is to correlate with emergent phenotypes. A caveat is that a recent study on methylmalonic acidemia (a genetic lesion downstream of that in PA) did not find disease normalization after inhibition of isoleucine and valine catabolism, arguing that their intermediates—including CoA esters—may not meaningfully contribute to phenotypes or that other propiogenic substrates (e.g. methionine) are responsible for dysfunction [28].

## Tracing histone modifications to phenotype

Given the difficulties in controlling propionyl-CoA experimentally, a more robust approach for seeking biological responses to raised propionyl-CoA is by means of inactivating or reducing PCC activity, which is why PA mice and human-derived PA stem cells are invaluable models. This approach comes with the problem of dissecting epigenetically mediated responses from the broader metabolic toxicity. Without selective tools for manipulating enzymes that catalyze PA-related histone marks, mapping onto biological responses remains difficult and correlative at best.

*In vitro* models provide tractable systems to follow responses to propionate and apply interventions that cannot practically or ethically be performed on animals. By regular medium changes, they also offer a means of alleviating toxin build-up to control confounders. In hiPSC-derived cardiomyocytes, a genetic reduction in PCC activity shifted fuel use from fatty acid oxidation towards glucose metabolism, which is significant because the heart harnesses 70% of its ATP from the former [29]. This metabolic shift is the inverse of insulin resistance in diabetes, where reliance on fatty acid oxidation increases; it is therefore of interest to test how a diabetic phenotype interacts with PA. Glucose oxidation is significantly less efficient in generating ATP compared with fatty acid oxidation, and a switch towards this could impair cardiac function, as seen in PA. However, this organ-level performance assessment requires a more complete model, which is why animal studies are indispensable.

Using animal models, histone propionylation was linked to cardiac dysfunction [6,30] and developmental deficits [17], consistent with the clinical presentation of PA. The extent to which histone PTMs can be assigned to specific downstream pathways depends on how faithfully the phenotypic readouts reflect the activity of those pathways. This relationship is typically more difficult to establish for neurological conditions compared with cardiac disease, which benefits from a range of imaging tools and well-mapped cascades. Notwithstanding the availability of measurement tools, establishing a role of epigenetics in metabolism-phenotype relationships is challenging because causality must be established beyond correlation, and confounders, such as a stress response or a non-histone route, must be excluded. Confidence in a role for histone PTMs in driving transcriptional response *in vivo* can build from evidence of comparable responses *in vitro*, which offers a more controlled environment. Park et al. compared transcriptional responses in PA mouse hearts against responses in isolated cardiomyocytes cultured in the presence of exogenous propionate [6]. This defined a subset of gene responses *in vivo* that could be replicated *in vitro*, thereby eliminating secondary responses, such as stress or adaptation. Responding genes included *Pde9a* and *Mme*, which also had proportional changes to histone propionylation and H3K27 acetylation marks at their promoters. Strikingly, the transcriptional responses were more pronounced in female PA mice, which was related to the higher degree of redox protection in male PA hearts attributed to histidyl dipeptides, a major class of antioxidant in the heart. The unexpected sex-dependence of phenotype provides further evidence for a role of histone PTMs, because the underlying PCC lesion is the same between male and female PA mice, yet differences emerge at the point of histone PTMs that transmit proportionally to cardiac dysfunction.

A reasonable critique of using PA mouse models to understand the impact of histone modifications is that the magnitude of biochemical disturbance is severe and may not relate to more physiological changes in propionyl-CoA. To that end, an alternative approach is to control propiogenic load through dietary interventions in animals with intact metabolic pathways. Yang et al. showed that reducing dietary branched-chain amino acids (BCAAs) blunted pressure-overload-induced H3K23 propionylation at promoters of genes involved in extracellular matrix remodeling and immune-cell signatures, and this was associated with reduced fibrosis and a more favorable cardiac stress response [30]. The BCAA-free diet also prevented the decline in electron transport chain gene expression that was associated with pressure overload. However, dietary propionate supplementation did not reverse the effect of BCAA restriction, arguing that the beneficial effect may be independent of propionylation; however, the authors did not measure cardiac propionyl-CoA levels, therefore it remains unclear whether propionate supplementation sufficiently modified cardiac metabolism.

The distinct phenotypes associated with propionyl-CoA buildup may reflect tissue specificity emerging at the biochemical, epigenetic, transcriptional, or translational levels. Insight into this can be borne from tissue-specific *Pcca* knockout models. Liver-specific ablation was found to recapitulate global knockout, highlighting the importance of hepatic metabolism in PA [7]. However, lysine propionylation was largely restricted to mitochondrial proteins, rather than histones. The present study shows that the epigenetic impact of a disease like PA may be highly tissue-specific and may therefore benefit from targeted treatments.

## Outlook for PA treatments

Current treatments for PA patients prioritize the correction of biochemical derangements; for example, dietary protein restriction to limit propiogenic substrate load, carnitine supplementation to enhance clearance, and carglumic acid administration to mitigate hyperammonemia. Since even aggressive dietary interventions do not adequately improve healthspan [31], it is plausible that some aspects of the disease are a diagnostic and therapeutic blind spot. One such aspect may be histone PTMs and altered transcriptional programs, which may be more resistant to normalization than blood-borne PA markers, such as 2-methylcitrate/citrate or propionylcarnitine/acetylcarnitine (C3/C2) ratios. However, the state of histone PTMs and their transcriptional responses are not currently part of clinical assessment due to methodological barriers and prohibitive costs.

Guidelines stipulate that restricting intake of the BCAAs valine and isoleucine is more efficacious than restricting methionine and threonine [32]. The efficacy of BCAA restriction in reducing abnormal propionylation was demonstrated in animals by Yang et al*.* [30], but whether this also applies to humans is unclear. Whilst biochemically rational, this strategy is practically difficult to implement in the long term, because propiogenic amino acids are required for protein synthesis; thus, over-restriction risks malnutrition, poor growth, net catabolism, and abnormal development. Indeed, recent nutritional studies complicate the assumption that precursor-free medical foods are metabolically neutral. Firstly, a greater dependence on protein substitutes was linked to weaker growth [31]. Secondly, intake of amino acid mixtures was found to potentially worsen nutritional and clinical scores and to produce knock-on effects on other amino acids, raising concerns about long-term consequences [32]. These observations argue that medical foods may not be metabolically neutral and that natural protein should be prioritized, if tolerated.

An alternative to dietary restriction is re-routing metabolism away from propionyl-CoA. The enzymes isobutyryl-CoA dehydrogenase (ACAD8) and 2-methylbutyryl-CoA dehydrogenase (ACADSB) process intermediates of valine and isoleucine, respectively, upstream of PCC but their inactivation does not produce PA-like symptoms. This striking observation suggests that upstream inhibition of BCAA oxidation may alleviate the propionyl-CoA load. Using a zebrafish model, Hong et al. demonstrated a protective effect of this strategy, pointing to the therapeutic potential of blocking ACAD8 and ACADSB in PA patients using small drugs [33]. A precedent for a successful small-molecule drug for controlling hyperammonemia in PA is carglumic acid. As a structural analogue of N-acetylglutamate, carglumic acid activates carbamoyl phosphate synthetase 1 and restores flux in the urea cycle [34].

While propiogenic substrate restriction is intuitively therapeutic for PA, other dietary components may improve patient outcomes when supplemented rather than omitted. For example, carnitine supplementation augments detoxification by conjugating excess propionyl groups as propionylcarnitine but whether this reduces histone propionylation has not been established. A more recently proposed experimental supplement is β-alanine, the rate-limiting substrate for synthesizing histidyl dipeptides in neurons and the heart [6]. By alleviating oxidative stress, histidyl dipeptide-enriched tissues could be better protected from the toxicity of propionyl-CoA but larger-scale preclinical studies are needed to establish this concept.

The interventions discussed thus far are grounded in the classical model of PA toxicity. Since these mechanisms are only partially understood, the scope for discovering new therapies is appealing. Reflecting that propionate and pyruvate converge onto the TCA cycle as anapleurotic pathways, Encarnacion et al. speculated whether the disturbance arising from decreased propionate entry at succinyl-CoA could be controlled by re-balancing flux through pyruvate entry [7]. In agreement with this proposal, the lethality of the liver-specific *Pcca* knockout was reversed by inactivating hepatic pyruvate carboxylate (*Pcx*). The *Pcca:Pcx* double-knockout rescued 2-methylcitrate levels, pointing to a key role of this intermediate in PA-associated toxicity. Their findings highlight *Pcx* as a new strategy for PA therapy and strengthen the case for seeking treatments that specifically suppress 2-methylcitrate build-up. However, simply blocking Pcx would be inadvisable because this pathway is essential in the urea cycle and gluconeogenesis; however, therapeutically rebalancing the TCA cycle is attainable through other means. Loss of *Pcx* is expected to increase acetyl-CoA, which would out-compete propionyl-CoA in *Pcca* knockout, restoring TCA activity and reducing 2-methylcitrate. This effect could be mimicked by increasing CoA production (hence acetyl-CoA) via pantothenate kinase (Pank) [35]. Since *Pcx* knockout reduces lysine acetylation in mitochondria [36], the double knockout may result in a fortuitous canceling-out of lysine modifications. The authors of the present study also highlight the importance of animal models in research on inherited metabolic disease, a matter for policymakers and grant funders to consider. Their *Pcca/Pcx* double knockout model produced a serendipitous phenotypic reversion *in vivo*, which led to a new avenue for understanding and treating PA. Such insight would not have been achievable without an animal model, where the most meaningful outcome—survival—can be measured directly.

## Conclusions and open questions

Propionyl-CoA is best described as a signaling metabolite that is both a toxic intermediate in PA and a substrate for histone acylation that can reconfigure transcription. Current literature supports a model in which propionyl-CoA disturbances generate tissue-specific histone propionylation and, indirectly, various other forms of acylation as the biochemical disturbance propagates across inter-connected pathways. This epigenetic mechanism may help explain why PA is associated with a sustained pathological remodeling of the heart. A case to include post-translational modification (PTM) biomarkers in the diagnosis and management of PA could be made on the principle that current biochemical readouts are not always predictive of disease severity. For example, 2-methylcitrate was only partially predictive of metabolic decompensation [37], indicating that conventional markers may not capture all dimensions of propionyl-CoA disturbances.

Several questions remain unresolved. How important are histone PTMs compared with mitochondrial dysfunction, oxidative stress, or non-histone modifications in driving PA pathology? How much of the chromatin response is driven by propionyl-CoA itself versus other intermediates that may impact alternative PTMs? Are there transferase enzymes that selectively favor propionylation? Is propionylation sufficiently distinct from other acylations to produce a unique transcriptional response? What underpins tissue-dependent responses? Is a reversal of histone PTMs essential for successful PA therapies? Should downstream markers of histone modifications, such as specific transcripts, be part of the clinical assessment of PA?

## Perspectives

- **Importance of the field:** Propionyl-CoA is emerging as a signaling metabolite that links metabolism to chromatin regulation through histone modifications. PA provides a disease model in which disturbed propionyl-CoA metabolism reveals the consequences of this metabolic/epigenetic interface.
- **Current thinking:** Excess propionyl-CoA appears to drive histone propionylation alongside broader changes in histone acylation, leading to tissue-specific transcriptional remodeling. Mouse models are useful in linking these epigenetic changes to cardiac and neurological phenotypes, but the relative contributions of histone propionylation, other PTMs, and secondary metabolic stress remain unclear.
- **Future directions:** Key goals are to define the causal contribution of histone propionylation, understand propionyl-CoA compartmentalization and tissue specificity, and identify enzymes or pathways that regulate propionylation. Integrating metabolomics, epigenomics, and functional phenotyping will determine whether these marks are biomarkers or therapeutic targets in PA and related metabolic disorders.

## Open Access

Open access for this article was enabled by the participation of the University of Oxford in an all-inclusive Read & Publish agreement with Portland Press and the Biochemical Society under an agreement with JISC.

## Abbreviations

BCAAs: branched-chain amino acids
HATs: histone acyl-transferases
HDACs: histone deacetylases
PTM: post-translational modification
PA: propionic acidemia
Pr-CoA: propionyl-CoA
PCC: propionyl-CoA carboxylase
TCA: tricarboxylic acid
